# Stress significantly increases mortality following a secondary bacterial respiratory infection

**DOI:** 10.1186/1297-9716-43-21

**Published:** 2012-03-21

**Authors:** Paul D Hodgson, Palok Aich, Joseph Stookey, Yurij Popowych, Andrew Potter, Lorne Babiuk, Philip J Griebel

**Affiliations:** 1 Vaccine & Infectious Disease Organization, 120 Veterinary Road, University of Saskatchewan, Saskatoon, SK, Canada S7N 5E3; 2 School of Biological Sciences, National Institute of Science Education and Research (NISER). IOP Campus, Bhubaneswar 751005 Odisha, India; 3 WCVM, 52 Campus Drive, University of Saskatchewan, Saskatoon, SK, Canada S7N 5E3; 4 University of Alberta, Edmonton, AB, Canada; 5 School of Public Health, University of Saskatchewan, Saskatoon, SK, Canada S7N 5E3

## Abstract

A variety of mechanisms contribute to the viral-bacterial synergy which results in fatal secondary bacterial respiratory infections. Epidemiological investigations have implicated physical and psychological stressors as factors contributing to the incidence and severity of respiratory infections and psychological stress alters host responses to experimental viral respiratory infections. The effect of stress on secondary bacterial respiratory infections has not, however, been investigated. A natural model of secondary bacterial respiratory infection in naive calves was used to determine if weaning and maternal separation (WMS) significantly altered mortality when compared to calves pre-adapted (PA) to this psychological stressor. Following weaning, calves were challenged with *Mannheimia haemolytica *four days after a primary bovine herpesvirus-1 (BHV-1) respiratory infection. Mortality doubled in WMS calves when compared to calves pre-adapted to weaning for two weeks prior to the viral respiratory infection. Similar results were observed in two independent experiments and fatal viral-bacterial synergy did not extend beyond the time of viral shedding. Virus shedding did not differ significantly between treatment groups but innate immune responses during viral infection, including IFN-γ secretion, the acute-phase inflammatory response, CD14 expression, and LPS-induced TNFα production, were significantly greater in WMS versus PA calves. These observations demonstrate that weaning and maternal separation at the time of a primary BHV-1 respiratory infection increased innate immune responses that correlated significantly with mortality following a secondary bacterial respiratory infection.

## Introduction

Primary viral respiratory infections have been recognized to be associated with an increased incidence and severity of secondary bacterial respiratory infections in humans [1,2] and respiratory infections in animals [3] for over 100 years. Secondary bacterial respiratory infections were frequently identified as the cause of death following influenza infections in humans and several mechanisms have been identified by which primary viral respiratory infections increase susceptibility to a secondary bacterial infection. Increased bacterial attachment and invasion are important contributing factors but viral-induced changes in leukocyte recruitment to the lung and the production of pro-inflammatory cytokines also contribute to lethal viral-bacterial synergy (reviewed in [4]). Thus, any factor that alters leukocyte recruitment and function in the lung during a primary viral infection may also impact the pathogenesis of a secondary bacterial respiratory infection.

Studies in mice demonstrated that specific psychological stressors either enhance [5] or inhibit [6,7] lung leukocyte recruitment and cytokine secretion following a primary influenza infection. Experimental influenza infections in humans revealed an association between psychological stress and increased production of IL-6, a pro-inflammatory cytokine [8]. Thus, psychological stressors significantly alter host responses during a primary viral respiratory infection. Epidemiological evidence links stress with an increased incidence and severity of respiratory infections in humans [9,10] and animals [11,12] but no experimental studies have been performed to determine if stress alters the viral-bacterial synergy resulting in fatal secondary bacterial infections in cattle.

Respiratory infections are a major cause of disease in all age groups of cattle and remain a major cause of economic losses in feedlot cattle [13]. Epidemiological studies have implicated a variety of stressors, including transportation, weaning, social re-organization, and dietary changes, with increased bovine respiratory disease (BRD) morbidity and mortality [11,12]. In a controlled study, duration of transportation was correlated with the magnitude of stress responses but transportation time had variable effects on morbidity and mortality in feedlot steers [14]. Neither transportation time nor distance were identified as significant risk factors correlating with fatal fibrinous pneumonia when studying young beef calves of unknown background and immune status [15]. This epidemiological study is consistent with biological observations that the stress of transportation has transient effects on alveolar macrophage [16] and blood leukocyte [17] function when assayed in vitro. In contrast, calves weaned immediately prior to being transported to a feedlot had a significantly increased incidence of undifferentiated BRD when compared to calves from a similar background but weaned 45 days earlier [18]. Suckling beef calves separated from their dams display increased vocalization and movement for 3-4 days after separation [19] and significant changes in serum protein, metabolite and elements were observed for 4 days post-weaning when comparing abrupt weaned and calves pre-adapted to weaning [20]. The study by Step et al. [18] suggests physiological responses to weaning are of sufficient duration to significantly alter host responses to a variety of pathogens which may cause BRD.

Fatal BRD is frequently associated with a primary viral infection followed by a secondary bacterial infection and multiple viral and bacterial pathogens have been implicated in this viral-bacterial synergy [21]. Bovine herpesvirurus-1 (BHV-1) and the Gram-negative bacterium, *Mannheimia haemolytica (M. haemolytica)*, are two important BRD pathogens for which reproducible experimental infection models have been developed [22,23]. Respiratory infection by either BHV-1 or *M. haemolytica *alone is rarely fatal but an aerosol challenge with *M. haemolytica *4 days after a BHV-1 infection causes a fatal secondary bacterial infection in 30-70% of calves [22]. This combined viral-bacterial infection model has been used to identify immune mechanisms contributing to viral-bacterial synergy following a primary BHV-1 infection [24,25]. Immune mechanisms implicated in fatal secondary *M. haemolytica *respiratory infections include altered alveolar macrophage function, altered polymorphonuclear leukocyte (PMN) function, decreased NK-cell activity, and increased production of pro-inflammatory cytokines. Increased production of pro-inflammatory cytokines following a primary BHV-1 infection is of particular interest in view of the pathology associated with an acute *M. haemolytica *respiratory infection [26]. Within hours of bacterial colonization of the lung there is a necrotizing inflammatory response that is dependent upon PMN recruitment to the lung [27] and increased production of pro-inflammatory cytokines such as interleukin (IL)-1, IL-8, and TNF-α [28]. Although many of the innate immune responses that occur during BHV-1 and *M. haemolytica *respiratory infections have been characterized, this model has not been used to determine whether the stress of weaning sufficiently alters viral-bacterial synergy to increase fatal BRD.

Weaning suckling beef calves immediately prior to placement in feedlots is a common industry practice in North Amercia and Step et al. [18] identified weaning as a significant factor contributing to the incidence of undifferentiated BRD despite the use of vaccines and antibiotics. Therefore, we hypothesized that the stress response to weaning could alter viral-bacterial synergy and enhance fatal BRD in naïve feedlot calves. To address this hypothesis, naïve calves were selected and either pre-adapted to the stress of weaning or weaned immediately prior to challenge with a primary BHV-1 infection and a secondary *M. haemolytica *infection. The controlled BRD infection model facilitated a comparative analysis of molecular and cellular responses which may contribute to enhanced fatal viral-bacterial synergy in weaned calves.

## Materials and methods

### Treatment groups

Female and castrated male, 5 to 6 month old, crossbred (Angus × Hereford), suckling calves were selected from a single herd. Calves were born in April and May and two independent BRD challenge experiments (Experiment 1 and 2) were performed during the last two weeks of October in consecutive years. Calves seronegative for BHV-1 and *M. haemolytica *were randomly assigned to one of two experimental groups (*n *= 10/group in Experiment 1; *n *= 20/group in Experiment 2). Group One (Pre-Adapted; PA) calves were separated from their dams, housed in a separate pen on the ranch, and fed free-choice, mixed brome and alfalfa hay and one kg/day whole oats for two weeks prior to BRD challenge. Group Two calves (WMS) grazed a mixed forage pasture and suckled their dams until weaning and separation from their dams immediately prior to being transported to the research facility. These calves were exposed to multiple stressors, including weaning, maternal separation (WMS) and transportation. The day prior to BHV-1 challenge, calves in the PA and WMS groups were collectively transported for 3.5 h and housed in a single pen at the VIDO Animal Research Facility. All calves had free-choice access to mixed brome and alfalfa hay and one kg/day whole oats throughout the BRD challenge period. The average weight of calves on arrival in both Experiment 1 and 2 was 232 kg with body weights ranging between 174 and 242 kg. There was no significant difference in average body weight when comparing WMS and PA groups within replicate experiments. Control calves (*n *= 10) for serum cortisol levels were age-matched, Hereford-Angus cross calves purchased from a separate herd. These calves were housed at VIDO and were fed mixed brome and alfalfa hay and one kg/day whole oats for one month prior to the experiment. Blood samples to isolate serum, PBMC and PMN were collected from the jugular vein prior to challenge with BHV-1 and daily thereafter until day 6 post-BHV-1 infection. Experiments were conducted according to the Guide to the Care and Use of Experimental Animals, provided by the Canadian Council on Animal Care and all experimental protocols were approved by University of Saskatchewan Animal Care Committee.

### Experimental infection

All calves were aerosol challenged with BHV-1 isolate 108 (5 × 10^7 ^pfu/animal) the day following transport. Animals were aerosol challenged with *M. haemolytica *strain PH45 (6 × 10^9 ^cfu/animal) either 4 days after BHV-1 infection (Experiment 1 (Group I and II) and Experiment 2 (Group I and half of Group II)) or 12 days after BHV-1 infection (Experiment 2 (Half of Group II)). Aerosol challenges were performed using an Ultra-Neb 99 nebulizer (model 099 HD, DeVilbiss, Somerset, PA). The strain and dose of BHV-1 and *M. haemolytica *used for challenge and the interval between primary viral infection and secondary bacterial challenge were previously optimized to induce clinical signs of viral respiratory disease in all calves with an expected mortality rate between 30-70% within 6 days after the secondary bacterial infection [22]. Shedding of infectious BHV-1 in nasal secretions was monitored daily by collecting nasal mucus, beginning the day of viral challenge. Briefly, sterile cotton swabs were used to collect nasal mucus and the swabs were immersed in one m1 MEM (GibcoBRL) prior to storing samples at -80°C. Virus in nasal secretions was quantified by plaque titration in microtiter plates with a neutralizing antibody overlay as previously described [29]. Following *M. haemolytica *challenge, animals were monitored every 6 h and animals unable to rise from recumbency were euthanized by intravenous injection of 100 mg sodium pentobarbital/kg body weight (Euthanyl, Biomeda MTC, Cambridge, Canada). The presence of *M. haemolytica *infection in animals with fatal respiratory disease was confirmed by bacterial culture of lung swabs collected during post-mortem examination.

### Gross pathology

Pathology scoring of entire lungs was performed to quantify grossly visible lung lesions known to be present following *M. haemolytica *infection and which are characterized by tissue consolidation, congestion and a fibrinous pleuropneumonia [26]. Scoring was performed by a clinical veterinarian blinded to treatment groups. Each lobe was visually examined with the lung positioned dorsal surface down and palpated before estimating the percentage of each lobe affected. Lungs lobes were given a value representing that lobe's percentage of total lung volume (Value A). These values are as follows: right cranial lobe = 6%; right posterior cranial lobe = 5%; right middle lobe = 7%; right caudal lobe = 35%; right intermediate lobe = 4%; left cranial lobe = 5%; left posterior cranial lobe = 6%; and left caudal lobe = 32%. The percentage of each lobe affected (Value B) was multiplied by the percentage volume for that lobe (Value A) and the sum of these products (A × B) for all lobes was recorded as a total lung score (out of 100%) for each animal.

### Clinical responses

Rectal temperature, body weight, and nasal lesions were monitored daily by a clinical veterinarian blinded to treatment groups. The experiment was terminated on the sixth day following bacterial challenge and all surviving animals treated with 10 mg tilmicosin/kg body weight (Micotil; Eli Lilly Canada Inc., Toronto, Canada). Nasal secretions for the analysis of IFN-α and IFN-γ were collected from all animal prior to BHV-1 challenge and 3 and 6 days post-BHV-1 challenge. Nasal secretions were collected by inserting a cotton tampon into the nostril for 20 min, absorbed fluid was expressed from the tampon, and stored at -80°C until IFN levels were analyzed by ELISA.

### ELISAs

Calves were screened for serum antibodies specific for BHV-1 and *M. haemolytica *using ELISA. Detection of BHV glycoprotein D (gD) specific antibodies was performed as previously described [30]. Titers below a 1/40 dilution of serum were considered negative. Serum antibodies specific for *M. haemolytica *leukotoxin were detected as previously described and titers below a 1/400 dilution of serum were considered negative [31]. The level of serum haptoglobin, a marker of acute inflammatory responses during bovine respiratory disease was determined using a bovine specific ELISA [32]. The concentration of IFN-α and IFN-γ in nasal secretions was determined by capture ELISA as previously described [33] and the concentration of TNF in PBMC culture supernatants was also determined by capture ELISA [34]. Serum cortisol levels were analyzed by Prairie Diagnostic Services (University of Saskatchewan) using the Immulite Cortisol Analyzer (Diagnostic Products Corporation, Los Angeles, CA, USA).

### Cell isolation and culture

Blood was collected from the jugular vein into 10 mL Vacutainers^® ^containing K_3 _EDTA 7.5% TriPotassium solution (Preanalytical Solutions, NJ, USA) and PBMC and PMN leukocytes were isolated as described previously [35]. PBMC were cultured in 96-well plates (Corning Inc., NY, USA) in a final volume of 200 μL/well AIM-V medium (InVitrogen Canada, Inc., Burlington, ON, Canada) supplemented with 5% fetal bovine serum (FBS; SeraCare, Milford, MA, USA). TNF production was assayed by plating 5 × 10^5 ^PBMC/well and stimulating cells with 100 ng/mL LPS (Sigma-Aldrich, St. Louis. MO). Culture supernatants were collected 24 h later and stored -80 °C until TNF levels were assayed.

### Gene expression analysis

Total RNA was isolated from 10 × 10^6 ^PBMC or PMN leukocytes using TRIZOL Reagent (Invitrogen Canada, Inc.) and further purified using RNeasy Mini-columns (Qiagen Inc. Canada, Mississauga, ON, Canada) as described previously [36]. RNA concentration was determined with an Agilent 2100 Bioanalyzer using RNA 6000 Nano kits (Agilent Technologies Canada Inc., Mississauga, ON, Canada). cDNA was prepared by reverse transcription of 500 ng total PBMC RNA or 100 ng of total PMN RNA using the SuperScript™ III Patinum^® ^Two-Step qRT-PCR Kit with SYBR^® ^Green (Invitrogen Canada Inc.) following the manufacturer's protocol. For each PCR reaction, 5 ng of cDNA was amplified with each primer set using the following parameters: 50 °C for 2 min to eliminate carry-over dUTP, then 45 cycles of 95 °C for 15 s; 55 °C for 30 s; and 72 °C for 30 s. Previously published oligo primers were used for the detection of bovine TLR4 [37], IL-1β [38] and IFN-γ [39]. Custom designed primers were designed using Clone Manager 7 (SciEd Software) and designed to span introns where possible (Table 1). Samples were amplified in duplicate using a Bio-Rad *iCycler *and a melt curve was completed following each PCR reaction to ensure fluorescence quantification was specific to the PCR amplified product. PCR products from custom designed primers were validated by sequence analysis using a Beckman CEQ2000XL. Amplification data are expressed as change in Cycle threshold (ΔCt) and calculated as follows: (ΔCt = Cycle threshold of Gene of Interest - Cycle threshold of GAPDH). A lower ΔCt value equates to more abundant transcript.

**Table 1:** Primer sequences for qRT-PCR amplification of cDNA

Bovine Gene	Accession Number	Primer Direction	Primer Sequence (5'-3')
*cd14*	NM_174008	**Forward**	5'-CACCACCCTCAGTCTCCGTAAC

		**Reverse**	5'-GCGAGTGTGCTTGGGCAATG

*il-10*	NM_174088	**Forward**	5'-GCTGTATCCACTTGCCAACC

		**Reverse**	5'-CCAGGTAACCCTTAAAGTCATCC

*2'5'oas*	NM_178108	**Forward**	5'-GTGCGAGAACCAGAGGAGAG

		**Reverse**	5'-TATTCTTATGCTTCATCTTACACAGTTG

*tnfα*	NM_173966	**Forward**	5'-GTAGCCGACATCAACTCTC

		**Reverse**	5'-AGGACCTGTGAGTAGATGAG

*gapdh*	AJ000039	**Forward**	5'-GGCAAGTTCAACGGCACAGTCAAG

		**Reverse**	5'-GTGCAGGAGGCATTGCTGACAATC

### Statistical analysis

Statistical analyses were performed using GraphPad Prism Version 6.10 software (GraphPad Software, Inc., San Diego, CA, USA). Differences in mortality were analyzed by comparing survival curves with a Gehan-Breslow-Wilcoxin test. Linear regression analyses were performed to determine if a significant correlation existed between lung damage versus day of death and secretion of IFN-α versus IFN-γ. Based on the assumption that data sets were not normally distributed, an unpaired t-test with Welch's correction was used for analyses of differences (days to death, daily body temperatures, serum haptoglobin, IFN-γ secretion, and gene expression) between treatment groups or when animals were grouped by disease outcome. Friedman one way analyses of variance (ANOVA) was used with Dunn's post-test when comparing changes in values over time (cortisol, haptoglobin, gene expression) within treatment groups. The Wilcoxon matched pairs test was used to assess significance of repeated measures of gene expression within groups.

## Results

### Abrupt weaning increases fatal secondary bacterial respiratory infection

**Figure 1:**
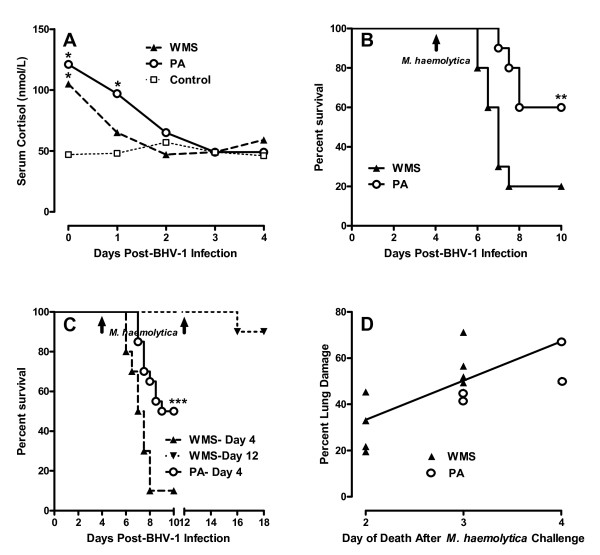
**Weaning and maternal separation (WMS) increases mortality following a secondary bacterial respiratory infection**. **(A) **Serum cortisol levels were measured prior to (Day 0) and for 4 days following BHV-1 infection. Data presented are median cortisol values for each group and WMS and pre-adapted calves (PA) were compared to non-transported (Control) calves. *p < 0.05. **(B) **Percent survival of WMS (*n *= 10) and PA (*n *= 10) calves following primary BHV-1 infection (Day 0) and secondary *M. haemolytica *infection (black arrow) four days later (Experiment #1). **p < 0.01. **(C) **Percent survival of PA (*n *= 20) calves following a primary BHV-1 infection (Day 0) and secondary *M. haemolytica *infection (black arrow) four days later. Percent survival of WMS calves following a primary BHV-1 infection (Day 0) and secondary *M. haemolytica *infection (black arrows) four days (*n *= 10) and 12 days (*n *= 10) later (Experiment #2). ***p < 0.001. **(D) **The percent lung damage correlated significantly (r^2 ^= 0.53, *p *< 0.01) with increased time to death following secondary bacterial challenge Values presented are for individual animals that died in both the WMS and PA groups.

Animals in both groups were transported the day prior to viral challenge and transportation induces cortisolemia [17]. Therefore, serum cortisol levels in WMS and PA calves were compared to Control calves that were not transported. Serum cortisol levels in both WMS (115.2 + 42.6; mean ± 1SD and PA (119.4 + 38.7; mean ± 1SD calves were significantly (*p *< 0.05) elevated the day after transport and prior to BHV-1 challenge when compared to the control group (47.6 + 22.7; mean ± 1SD. Serum cortisol levels in the WMS group returned to a level similar to Control calves within 24 h and within 48 h for the PA group (Figure 1a). Therefore, transportation was a common stressor in both treatment groups and at no time was there a significant difference in cortisol levels when comparing PA and WMS groups.

In Experiment 1, mortality was significantly (*p *< 0.01) greater in WMS (8/10 calves) than PA calves (4/10 calves) following the secondary bacterial infection (Figure 1b). The increased mortality in the WMS group was re-evaluated in a second independent experiment and the duration of fatal viral-bacterial synergy was further examined in the WMS group. Therefore, half the WMS calves (*n *= 10) were challenged with *M. haemolytica *4 days and half were challenged 12 days post-BHV-1 infection when viral shedding has stopped [23]. *M. haemolytica *challenge four days after BHV-1 infection resulted in significantly (*P *< 0.001) increased mortality in the WMS (9/10 calves) versus the PA (10/20 calves) group. There was, however, a marked reduction in fatal viral-bacterial synergy (1/10 calves) when WMS calves were challenged with *M. haemolytica *12 days after BHV-1 infection (Figure 1c). Therefore, the effect of weaning and maternal separation on susceptibility to fatal BRD did not extend beyond the time of virus shedding.

The interval between *M. haemolytica *challenge and onset of mortality was also significantly shorter in the WMS group than the PA group. In both experiments, survival time following secondary bacterial challenge was significantly (*p *< 0.007) reduced in the WMS group (2.7 days ± 0.54 days; mean ± 1SD) versus the PA group (3.6 days ± 0.46 days; mean ± 1SD) and there was a positive correlation (r^2 ^= 0.53; *p *< 0.01) between increased lung pathology as survival time was prolonged (Figure 1d). *M. haemolytica *was cultured from the lungs of all animals with fatal respiratory disease but reduced lung pathology in calves with acute mortality suggested increased stress in the WMS group did not directly increase bacterial-induced lung pathology. This observation lead to an examination of systemic innate immune responses following viral infection in Experiment 2 to determine if host responses to gram negative bacteria were significantly altered.

### Stress alters host responses to viral infection but not viral shedding

**Figure 2:**
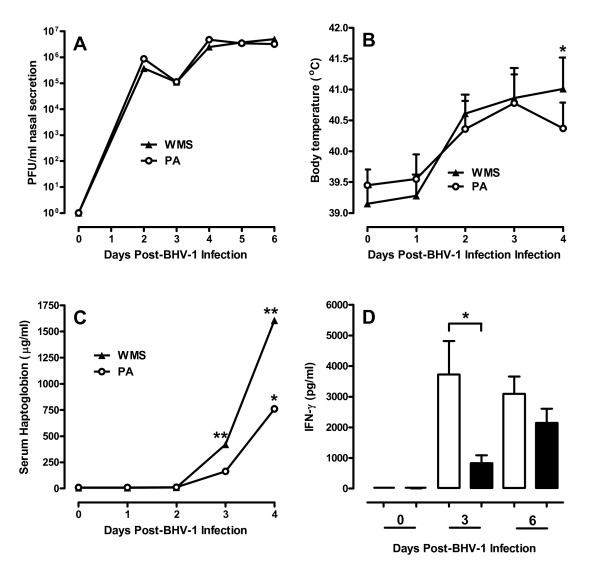
**Virus shedding and clinical responses following BHV-1 infection**. **(A) **Virus shedding in nasal secretions is expressed as plaque forming units (PFU) per ml of nasal secretion collected. Data presented are median values for weaned and maternal separation (WMS) and pre-adapted (PA) calves. **(B) **Rectal body temperatures of animals presented as mean + 1SD of values for WMS and PA calves. Values were compared between groups for each day post-BHV-1 infection. *p < 0.05. **(C) **Serum haptoglobin levels were analyzed daily within group following BHV-1 infection and data presented are median values for the WMS and PA groups. *p < 0.05; **p < 0.01. **(D) **IFN-γ levels in nasal secretions were quantified by capture ELISA and samples were collected prior to BHV-1 infection (Day 0) and 3 and 6 days post-infection. Day 6 was also 2 days after *M. haemolytica *challenge. Data presented are mean + 1SD for the WMS (open bars) and PA (solid bars) calves (*n *= 10/group). *p < 0.05.

Virus shedding in nasal secretions was monitored but no significant difference in viral titres was observed when WMS and PA calves were compared (Figure 2a). Fever, weight loss, and haptoglobin were measured to determine if weaning and maternal separation altered clinical responses following BHV-1 infection despite similar levels of virus shedding. There was a significant (*p *< 0.05) but similar reduction in body weight on day 4 post-BHV-1 infection in both WMS (-11.9 kg + 6.2 kg; mean ± 1 SD) and PA calves (-11.0 kg + 7.8 kg; mean ± 1 SD). There was, however, a significant (*p *< 0.05) divergence in body temperature on day 4 post-BHV-1 infection and prior to *M. haemolytica *infection (Figure 2b) when comparing the WMS (41.0 ± 0.55 °C; mean ± 1SD) and PA (40.3 ± 0.42 °C; mean ± 1SD) calves. Furthermore, only the WMS group displayed significantly elevated serum haptoglobin levels on both day 3 and 4 post-BHV-1 infection (Figure 2c). Fever and serum haptoglobin are acute-phase responses induced by a variety of cytokines, including IL-1, IL-6 and IFN-γ [32,33,40]. Therefore, IFN-α and - γ levels in nasal secretions were measured to quantify host response to viral infection. IFN-γ levels were significantly greater in WMS (3721 ± 740 pg/mL; mean ± 1SD than PA (828 ± 642 pg/mL; mean ± 1SD) calves 3 days after viral infection (Figure 2d). IFN-α was also present in nasal secretions on day 3 post-BHV-1 infection but at a lower level than IFN-γ. There was no significant difference when comparing IFN-α levels in WMS (1571 ± 1420 pg/mL; mean ± 1SD) and PA (809 ± 912 pg/mL; mean ± 1SD) calves but there was a significant correlation (*p *< 0.01) between IFN-α and -γ levels within individual animals (data not shown).

### Correlation of pro-inflammatory responses with stress and disease outcome

**Figure 3:**
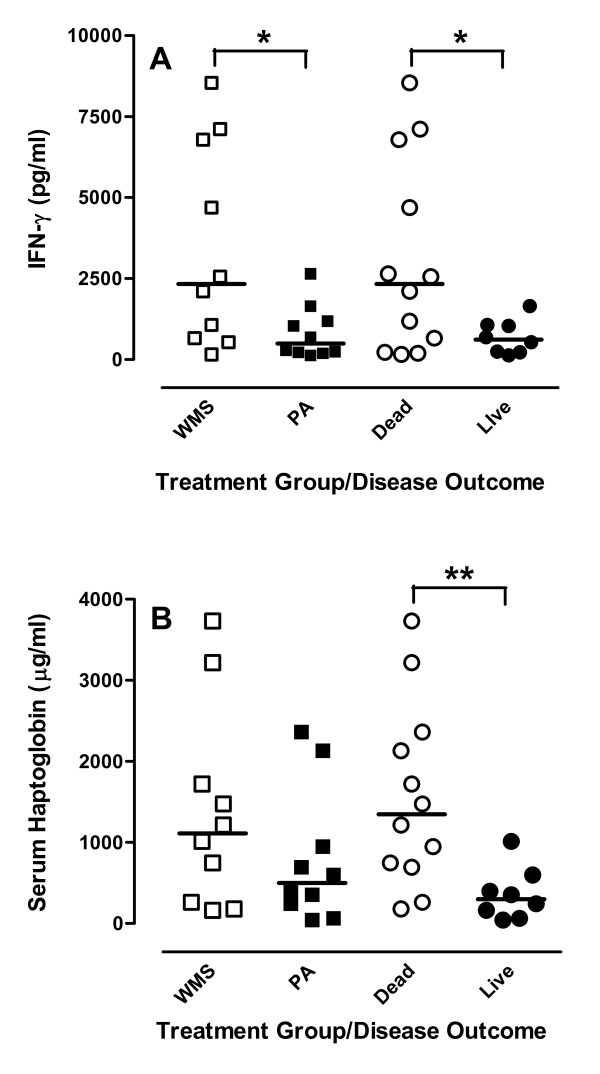
**Stress alters the antiviral response but not the acute-phase response responses**. **(A) **IFN-γ levels in nasal secretions on day 3 post-BHV-1 infection were compared between weaned and maternal separation (WMS) and pre-adapted (PA) calves (*n *= 10/group) and data was also sorted for calves that died (Dead; *n *= 12) or survived (Day 0; *n *= 8) following *M. haemolytica *infection. Data presented are values for individual animals and horizontal bars represent median values for each group. *p < 0.05. **(B) **Serum haptoglobin levels determined by capture ELISA were compared between WMS and PA calves (*n *= 10/group) and data was also sorted for calves that died (Dead; *n *= 12) or survived (Day 0; *n *= 8) following *M. haemolytica *infection. Data presented are values for individual animals and horizontal bars represent median values for each group. **p < 0.01.

Inflammatory responses to BHV-1 infection were analyzed on the basis of both stress treatment and BRD outcome. IFN-γ levels in nasal secretions on day 3 post-BHV-1 infection were significantly elevated when comparing calves that died versus survived secondary bacterial infection (Figure 3a). In contrast, serum haptoglobin levels at the time of bacterial challenge were not significantly different between WMS and PC groups but were significantly associated with disease outcome (Figure 3b). 2'5'-OAS expression, an IFN-induced antiviral response, was significantly elevated in PBMC of all calves following BHV-1 infection, confirming IFN produced at the site of viral infection had systemic effects (Table 2). TNF-α and IL-10 expression were also significantly elevated in PBMCs isolated from calves on day 4 post-BHV-1 infection but these responses were similar when comparing WMS and PA calves (Table 2). Resorting gene expression data by disease outcome revealed similar changes in both OAS and TNF-α expression in PBMCs (Table 3). There was, however, a significantly higher level of IL-10 expression following BHV-1 infection in animals that died following secondary bacterial infection (Table 3).

**Table 2:** Effect of stress on gene expression following BHV-1 infection

	WMS^1^		PA^1^	
**Gene**	**Day 0^2^**	**Day 4^3^**	**Day 0**	**Day 4**

2'5'OAS	3.3 ± 2.8^4^	0.9 ± 1.2*	2.7 ± 2.1	0.0 ± 1.5**

IL-β	6.8 ± 0.9	6.7 ± 0.8	7.0 ± 0.8	6.7 ± 0.6

TNF-α	6.1 ± 0.4	4.8 ± 0.7**	6.0 ± 0.4	4.9 ± 0.3**

IL-10	8.2 ± 0.8	5.7 ± 0.6**	8.1 ± 0.6	6.1 ± 0.3**

CD14	4.3 ± 0.7	3.4 ± 1.0*	4.2 ± 0.4	3.8 ± 0.7

TLR4	4.1 ± 0.9	3.2 ± 0.9*	4.0 ± 0.4	3.6 ± 0.6*

^1^Weaned and maternal separation (WMS) and pre-adapted (PA) calves (*n *= 10/group).

^2^PBMC isolated from calves prior to BHV-1 infection.

^3^PBMC isolated from calves 4 days post-BHV-1 infection and prior to *M. haemolytica *infection.

^4^Data presented are mean ΔCt ± 1SD with lower Ct value corresponding to greater transcript abundance.

*p < 0.05 when Day 4 compared to Day 0 within each group; **P < 0.01 when Day 4 compared to Day 0 within each group.

**Table 3:** Association between gene expression and BRD outcome

Dead^1^			Alive^1^	
**Gene**	**Day 0^2^**	**Day 4^3^**	**Day 0**	**Day 4**

2'5'OAS	2.5 ± 1.1^4^	0.0 ± 1.2**	3.6 ± 2.9	1.2 ± 1.5*

IL-β	6.9 ± 0.8	6.6 ± 0.8	7.0 ± 0.9	6.9 ± 0.5

TNF-α	6.0 ± 0.4	4.8 ± 0.5**	6.0 ± 0.5	5.1 ± 0.5*

IL-10	8.0 ± 0.8	5.6 ± 0.4**	8.4 ± 0.5	6.4 ± 0.3*§§

CD14	4.3 ± 0.7	3.1 ± 0.6**	4.2 ± 0.4	4.3 ± 0.7§§

TLR4	4.0 ± 0.8	2.9 ± 0.6**	4.2 ± 0.5	4.0 ± 0.6§§

^1^Data sorted for animals that either died (Dead) or survived (Alive) following *M. haemolytica *aerosol challenge.

^2^PBMC isolated from calves prior to BHV-1 infection.

^3^PBMC isolated from calves 4 days post-BHV-1 infection prior to *M. haemolytica *infection.

^4^Data presented are mean ΔC ± 1SD with lower Ct value corresponding to greater transcript abundance.

*P < 0.05 when Day 4 compared to Day 0 within group; **P < 0.01 when Day 4 compared to Day 0 within group; §§ *P *< 0.01 when Day 4 values compared across groups.

### Stress increases PBMC capacity to respond to gram-negative bacteria

IFN-γ enhances expression of TLR4 complex components [41], therefore we investigated whether the stress-induced increased in IFN-γ expression (Figure 3a) was associated with an altered capacity to respond to LPS. CD14 and TLR4 gene expression were analyzed in PBMC and PMN. TLR4 expression level (ΔCt expressed as mean ± 1 SD; *n *= 10) in PMNs prior to BHV-1 infection (Day 0) were 0.40 ± 0.62 (WMS) and 0.15 ± 0.85 (PA). During BHV-1 infection (Day 4), TLR4 expression levels in PMNs remained unchanged at 0.35 ± 0.44 (WMS) and 0.20 ± 0.70 (PA). CD14 expression level (ΔCt expressed as mean ± 1 SD; *n *= 10) in PMNs prior to BHV-1 infection (Day 0) were 5.8 ± 0.4 (WMS) and 5.1 ± 0.9 (PA) and CD14 expression levels also remain unchanged during BHV-1 infection (Day 4) at 6.1 ± 0.6 (WMS) and 6.2 ± 0.5 (PA). Therefore, further analyses of CD14 and TLR expression were restricted to PBMC and included a functional analysis of PBMC responses to LPS stimulation.

**Figure 4:**
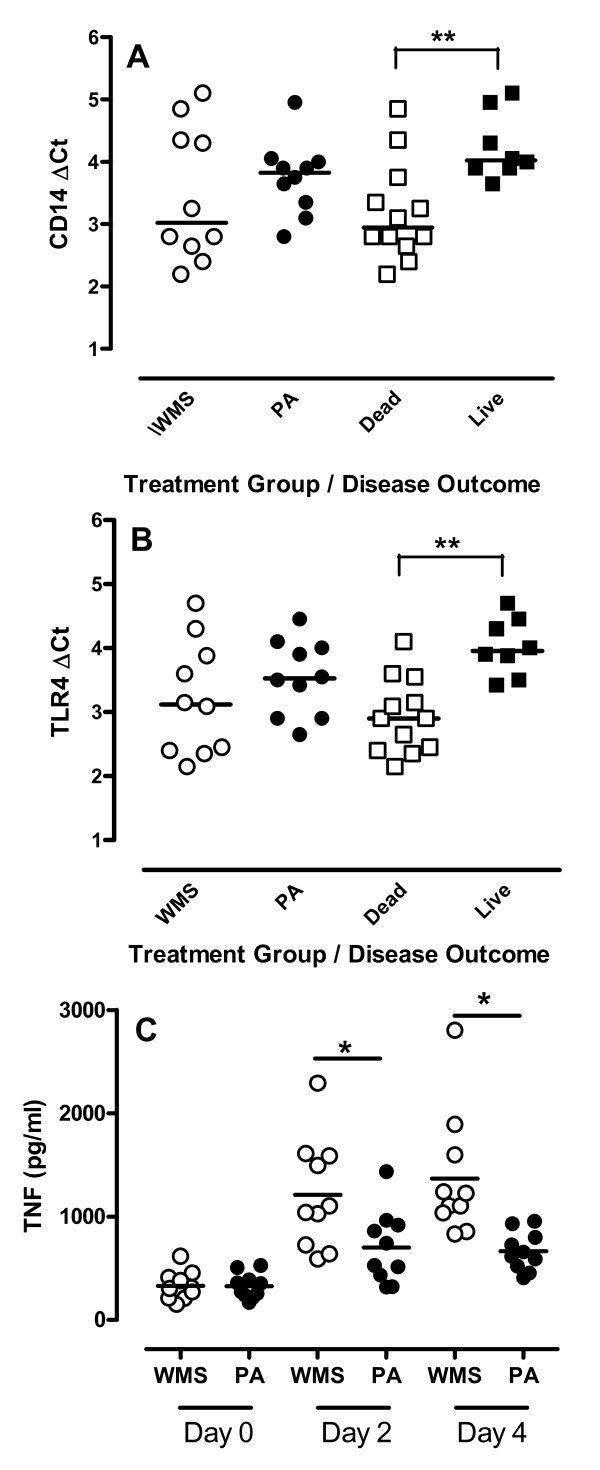
**CD14 and TLR4 expression in PBMC and LPS-responsiveness following BHV-1 infection**. **(A) **qRT-PCR analysis of CD14 expression levels (ΔCt) in PBMC on day 4 post-BHV-1 infection. Data presented are values for individual animals in weaned and maternal separation (WMS) and pre-adapted (PA) groups (*n *= 10/group) and horizontal bars represent median values for each group. A lower ΔCt corresponds to greater mRNA abundance. Data was also sorted for calves that died (Dead; *n *= 12) or survived (Day 0; *n *= 8) following *M. haemolytica *infection. **(B) **qRT-PCR analysis of TLR4 expression levels (ΔCt) in PBMC on day 4 post-BHV-1 infection. Data presented are values for individual animals in the WMS and PA groups (*n *= 10/group) and horizontal bars represent median values for each group. Data was also sorted for calves that died (Dead; *n *= 12) or survived (Day 0; *n *= 8) following *M. haemolytica *infection. **(C) **TNF-α secretion by PBMC was measured 24 h after LPS 20 stimulation. PBMC were isolated prior to BHV-1 infection (Day 0) and 2 and 4 days post-BHV-1 infection. Data presented are values for individual animals in the WMS and PA groups (*n *= 10/group) and horizontal bars represent median values for each group. *p < 0.05; **p < 0.01.

Quantitative RT-PCR analysis revealed significantly (*p *< 0.05) increased CD14 and TLR4 expression in PBMC isolated from WMS calves but only TLR4 expression was significantly (*p *< 0.05) increased in PBMC from PA calves on day 4 post-BHV-1 infection (Table 2). There was not, however, a significant difference in either CD14 (Figure 4a) or TLR4 (Figure 4b) expression levels in PBMC when WMS and PA groups were compared. In contrast, significant (*p *< 0.01) differences in both CD14 (Figure 4a) and TLR4 expression (Figure 4b) were apparent when data was sorted by disease outcome. Animals dying from secondary bacterial respiratory infection had significantly (*p *< 0.01) higher CD14 and TLR4 expression levels at the time of bacterial challenge. Furthermore, only animals succumbing to bacterial infection displayed a significant (*p *< 0.01) increase in both CD14 and TLR expression levels when comparing pre-infection (day 0) versus the time of bacterial challenge on 4 day post-BHV-1 infection (Table 3).

PBMC responses to LPS stimulation were analyzed by measuring TNF secretion in the culture supernatant of PBMC isolated from animals in the second BHV-1 and *M. haemolytica *challenge experiment. PBMC isolated from WMS calves but not PA calves displayed significantly (*p *< 0.05) increased TNF secretion on day 2 and 4 post-BHV-1 infection (Figure 4c) and TNF secretion by the WMS group was significantly greater than the PA group on day 2 and 4 post-BHV-1 infection.

## Discussion

Epidemiological studies have correlated a variety of stressors with an increased risk of fatal secondary bacterial respiratory infections in humans [10] and respiratory disease in animals [11,12]. Contradictory evidence has been reported regarding the contribution of transport stress to undifferentiated BRD in feedlot calves [14,15] but weaning was significantly correlated with an increased incidence of undifferentiated BRD [18]. The impact of weaning on mortality was minimal in this study, however, due to concurrent vaccination and treatment with antibiotics. The present investigation determined that stress from weaning and maternal separation (WMS) altered fatal viral-bacterial synergy in naïve calves when weaning was initiated at the time of a primary BHV-1 infection. This coincidence of WMS stress and a primary BHV-1 infection in naïve calves significantly increased mortality following a secondary *M. haemolytica *respiratory infection in two independent trials with only 10-20% of calves surviving the secondary bacterial challenge (Figure 1b and 1c). Furthermore, the effect of WMS on the fatal viral-bacterial synergy was of limited duration with only 10% mortality when the secondary bacterial infection was initiated 12 days after virus infection and weaning (Figure 1c). A previous investigation reported that calves weaned immediately before transport to a feedlot developed significantly more undifferentiated BRD when compared to calves adapted to weaning for 45 days [18]. The current observations (Figure 1c) indicate, however, that the effect of WMS stress on viral-bacterial synergy is of short duration. Therefore, management interventions which either mitigate stress responses, minimize exposure to respiratory pathogens, or enhance specific immunity to respiratory pathogens may be of greatest value within the first four days after weaning.

The rapid onset of death with reduced lung pathology (Figure 1) suggested that both systemic and local responses in the lung may have contributed to increased mortality in WMS calves. Previous analysis of stress responses in mice demonstrated that individual stressors may either enhance [5,6] or inhibit [6,7] local responses in the lung following a viral respiratory infection but the effect of stress on systemic responses was not analyzed. We used molecular and cellular analyses to determine if WMS had significant effects on innate immune responses to viral infection and observed a significant increase in both CD14 expression (Table 2) and LPS responsiveness during viral infection and prior to secondary bacterial challenge (Figure 4c). Therefore, WMS stress significantly increased innate immune responses during BHV-1 infection (Figure 3a). These increased innate immune responses are directly linked to the recognition of *M. haemolytica *infection through lipopolysaccharide (LPS) [42,43]. There is increasing evidence that respiratory viral infections can modulate expression of receptors involved in the recognition of LPS. TLR4 expression was increased on blood monocytes following human respiratory syncytial virus (RSV) infection of young children [44] and Porcine Reproductive-Respiratory Syndrome (PRRS) virus induced increased expression of both CD14 and LPS binding protein in the lung [45]. In agreement with these observations, our transcriptional analyses revealed that BHV-1 infection of WMS calves increased PBMC expression of both CD14 and TLR4 and increased expression of these receptors correlated significantly (*p *< 0.01) with fatal secondary bacterial infections (Table 3). Thus, modulating expression of TLRs and associated adaptor molecules by primary viral infections may be a general mechanism of viral-bacterial synergy. In support of this conclusion, we also observed increased expression of TLR2, the receptor for peptidoglycans, following BHV-1 infection (data not shown). Thus, primary viral respiratory infections could potentially enhance pro-inflammatory responses to both Gram-negative and Gram-positive bacterial infections. It is also interesting to note that increased expression of TLR4 by itself in PA calves was not sufficient to significantly increase LPS-induced TNF secretion (Figure 4c). Therefore, increased responsiveness to LPS may depend on an up-regulation of all components of the TLR4 signaling complex.

Identifying the mechanism(s) by which specific stressors, such as WMS, enhance TLR expression and function during viral infections may increase our understanding of how individual stressors increases mortality following a secondary bacterial respiratory infection. BHV-1 is a potent inducer of IFN-α and IFN-γ secretion in the upper respiratory tract [33,46] and both cytokines have been identified as mediators of LPS-sensitization following viral infection [41,47]. Although earlier studies did not identify the mechanism by which IFN induce LPS-sensitization, a recent investigation revealed that IFN-γ increases expression of both TLR4 and CD14 and enhances LPS-induced responses of human macrophages [41]. Thus, significant correlations between mortality and IFN-γ secretion, mortality and TLR4 expression, and mortality and CD14 expression is consistent with a causal relationship between weaning enhanced anti-viral responses and increased viral-bacterial synergy. A previous analysis of blood leukocyte populations during BHV-1 infection revealed that the number of blood monocytes remain relatively constant during infection [46]. Thus, increased expression of TLR4 and CD14 in PBMCs cannot be explained by a simple increase in monocyte frequency following viral infection. A possible link between IFN secretion at the site of viral infection and altered gene expression in PBMC is also supported by increased 2'5' OAS gene expression in PBMC following viral infection (Table 1). However, co-production of both IFN-α and IFN-γ following BHV-1 infected calves makes it difficult to state whether one or both of these IFNs contributed to altered TLR4 and CD14 expression and function.

If IFN contributes to the enhanced mortality observed with stress then the question arises as to how stress enhances IFN-γ production in WMS calves (Figure 2d). Glucocorticoid responses to stress can inhibit cytokine production [48] and may have an effect on BHV-1 replication [49]. There were, however, no significant differences in either cortisol level (Figure 1a) or virus shedding (Figure 2a) when comparing WMS and PA calves. Therefore, there was no apparent connection between cortisol production and altered viral-bacterial synergy in WMS calves. Furthermore, gene expression analysis revealed IFN-γ expression did not change in PBMC of either experimental groups following viral infection (data not shown). This observation may be consistent with a previous report that BHV-1 infection did not activate NK cell activity in blood, but resulted in the rapid recruitment of active NK cells to the site of infection [50]. Enhanced recruitment and activation of NK cells in the respiratory tract of WMS calves may be one mechanism by which stress could increase IFN-γ secretion. Specific psychological stressors, such as restraint, can inhibit leukocyte migration to the murine lung [7], but social re-organization was shown to increase leukocyte migration into the lung and immunopathology [6]. Thus, it is conceivable that the combination of stressors represented by weaning and maternal separation enhanced leukocyte recruitment to and IFN production at the site of viral infection. Increased IFN at the site of BHV-1 replication would also be consistent with the similar levels of BHV-1 replication in both experimental groups (Figure 2a) since BHV-1 is highly resistant to the antiviral effects of IFN [46].

It was hypothesized previously that pro-inflammatory responses induced by a primary BHV-1 infection contribute to the viral-bacterial synergy of a fatal *M. haemolytica *infection [25]. Elevated body temperature and serum haptoglobin levels in the WMS group (Figure 2) may be explained by increased IFN production since expression of pro-inflammatory cytokines, such as IL-1 and TNF-α, were not significantly different in WMS versus PA calves (Table 2) or calves that died versus survived following secondary bacterial infection (Table 3). Viral infection did, however, induce significantly increased IL-10 expression levels in the PBMC of animals with fatal viral-bacterial synergy (Table 2). IL-10 can induce IFN-γ secretion by NK cells [51] and increased IL-10 may provide positive feedback to increase IFN-γ secretion during viral infection (Figure 3c). Thus, increased mortality in WMS calves may involve dys-regulation of the pro-inflammatory response. The more prolonged elevation of serum cortisol in the PA group (Figure 1a) may be another factor contributing to the lower pro-inflammatory responses in this group since corticosteroids have potent anti-inflammatory activity [48]. It should also be noted, however, that both treatment groups in the present experiment and previous experiment [18] experienced multiple stressors, including transportation, social mixing, altered environment, and restraint during sample collection or treatment. Therefore, it remains to be determined if weaning and maternal separation, in the absence of any other stressors, is sufficient to significantly increase viral-bacterial synergy. Specific stressors or combinations of stressors may influence the activity of either the hypothalamus-pituitary adrenal axis or the sympathetic adrenal medullary axis in distinct ways and have very different effects on the immune response to respiratory viral infections [5-7]. It may be that one or more concurrent stressors were required to induce stress responses of sufficient magnitude or duration to significantly (*p *< 0.001) increased BRD mortality (Figure 1). The BHV-1 and *M. haemolytica *infection model provides a system to begin investigating the temporal relationship between individual or combined stressors on viral-bacterial synergy and to identify mechanisms by which specific stressors alter susceptibility to BRD. This would be the first step in identifying behavior modification protocols or therapeutic agents that effectively mitigate the effects of stress on disease susceptibility.

## Abbreviations

BHV-1: Bovine herpesvirus-1; BRD: Bovine respiratory disease; IFN-γ: interferon-gamma; 2'5' OAS: 2'5' oligoadenylate synthetase; PA: Pre-adapted; PBMC: peripheral blood mononuclear cells; PMN: Polymorphonuclear leukocytes; WMS: Weaned and maternal separation.

## Competing interests

The authors declare that they have no competing interests.

## Authors' contributions

PH designed primers, performed gene expression analysis and assisted in drafting the manuscript. PA assisted in primer design and gene expression analysis and contributed to study design. JS contributed to study concept and design and manuscript preparation. YP isolated blood leukocytes, archived samples, and performed leukocyte function assays. AP and LP contributed to study design and manuscript preparation. PG contributed to the concept and design of the study, conducted animal trials, collected and analyzed data, and completed manuscript preparation. All authors read and approved the final manuscript.
